# Mining Novel Constitutive Promoter Elements in Soil Metagenomic Libraries in *Escherichia coli*

**DOI:** 10.3389/fmicb.2018.01344

**Published:** 2018-06-20

**Authors:** Cauã A. Westmann, Luana de Fátima Alves, Rafael Silva-Rocha, María-Eugenia Guazzaroni

**Affiliations:** ^1^Department of Cellular and Molecular Biology, FMRP, University of São Paulo, Ribeirão Preto, Brazil; ^2^Department of Biology, FFCLRP, University of São Paulo, Ribeirão Preto, Brazil; ^3^Department of Biochemistry, FMRP, University of São Paulo, Ribeirão Preto, Brazil

**Keywords:** functional metagenomics, bi-directional reporter, constitutive promoters, synthetic biology, high-throughput screening

## Abstract

Although functional metagenomics has been widely employed for the discovery of genes relevant to biotechnology and biomedicine, its potential for assessing the diversity of transcriptional regulatory elements of microbial communities has remained poorly explored. Here, we experimentally mined novel constitutive promoter sequences in metagenomic libraries by combining a bi-directional reporter vector, high-throughput fluorescence assays and predictive computational methods. Through the expression profiling of fluorescent clones from two independent soil sample libraries, we have analyzed the regulatory dynamics of 260 clones with candidate promoters as a set of active metagenomic promoters in the host *Escherichia coli*. Through an in-depth analysis of selected clones, we were able to further explore the architecture of metagenomic fragments and to report the presence of multiple promoters per fragment with a dominant promoter driving the expression profile. These approaches resulted in the identification of 33 novel active promoters from metagenomic DNA originated from very diverse phylogenetic groups. The *in silico* and *in vivo* analysis of these individual promoters allowed the generation of a constitutive promoter consensus for exogenous sequences recognizable by *E. coli* in metagenomic studies. The results presented here demonstrates the potential of functional metagenomics for exploring environmental bacterial communities as a source of novel regulatory genetic parts to expand the toolbox for microbial engineering.

## Introduction

The study of prokaryotic transcriptional regulation is essential for understanding the molecular mechanisms underlying decision-making processes in microorganisms (Ishihama, 2010), comprising populational, ecological and pathogenic behaviors. The activity of most bacterial promoters is usually dependent on the combined action of transcription factors and sigma factors in response to multiple environmental stimuli (Browning and Busby, 2016). For instance, in *Escherichia coli*, the compilation of decades of experimental data indicate that ~50% of its promoters are under the control of a single specific regulator, while all other genes are regulated by at least two transcription factors (Gama-Castro et al., 2016). Moreover, the recent development of experimental and large-scale sequencing techniques, together with powerful computational approaches have allowed both the discovery of insightful information about other bacterial transcriptional systems and the development of novel approaches for studying them n higher depth (Shen-Orr et al., 2002; Martínez-Antonio and Collado-Vides, 2003; Covert et al., 2004; Shimada et al., 2005). However, despite technical innovations, most of the studies are still centered on *E. coli*, a single bacterial species among at least 30,000 other already sequenced (Land et al., 2015), in an estimated total of 1 trillion species (Locey and Lennon, 2016).

With the advent of Metagenomics (Handelsman et al., 1998), the exploration of unculturable bacteria (~99% of a bacterial community (Amann et al., 1995) widely expanded genomic information, providing resourceful data about populational structures and genetic diversity in a myriad of environmental samples (Torsvik and Øvreås, 2002; Venter, 2004; Tringe, 2005). Two main approaches are commonly adopted for those metagenomic studies (Singh et al., 2009): the sequence-based metagenomic approach, which relies on massive sequencing of metagenomic DNA and powerful bioinformatics tools for extracting information from the metagenomic sequences; and functional metagenomics (Cowan et al., 2005; Li and Qin, 2005), which directly explores the functionality of enzymes and other structural elements through a wide range of stress/substrate/product-based assays (Uchiyama et al., 2005; Uchiyama and Miyazaki, 2010; Guazzaroni et al., 2013). In this context, although a large number of genes/ORFs has been discovered through the previously described approaches, the detection of novel bacterial regulatory elements using high-throughput technologies has been poorly explored, presenting so far a single well-defined method for the discovery of substrate-inducible regulatory sequences—SIGEX (Uchiyama et al., 2005)—and a direct assay for prospecting promoters for industrial applications (Han et al., 2008). This scarce number of methodologies is directly related to the biased search toward novel enzymatic activities and to a lack of both experimental and computational tools for finding and validating promoter sequences in metagenomic libraries (Guazzaroni et al., 2015).

Unraveling novel bacterial promoters is essential for understanding the regulatory diversity of microorganisms, addressing important questions, such as the abundance of both constitutive and inducible elements in a metagenomic library, the bottlenecks regarding host choices (i.e., the constrains limiting the diversity of exogenous promoters that can be recognized by different hosts) and the correlation between promoter strength, transcriptional noise and the functional role of the regulated gene/operon (Ekkers et al., 2012; Silander et al., 2012; Guazzaroni et al., 2015; Vester et al., 2015). Furthermore, prospecting, and characterizing novel promoters is crucial for expanding the current Synthetic Biology toolbox and generating novel biotechnological applications as there is a high demand for constitutive and inducible promoters responding to process-specific parameters (Uchiyama et al., 2005; Silva-Rocha and de Lorenzo, 2008; Boyle and Silver, 2009; Blount et al., 2012; Guazzaroni et al., 2015).

In this context, the most common strategy for prospecting promoters is the usage of trap-vectors, which consist in transcriptional fusions between DNA fragments and a reporter gene. This method has been widely employed for assessing promoters in genomic DNA (Kubota et al., 1991; Dunn and Handelsman, 1999; Lu et al., 2004; Chen et al., 2007), however its application in metagenomic DNA fragments has remained poorly explored (Uchiyama et al., 2005; Han et al., 2008). Furthermore, most adopted promoter trap-systems are unidirectional, while bacterial genomes present a large variation in the percentage of their leading-strand genes, ranging from ~45 to ~90% (Mao et al., 2012, 2015), suggesting that a bi-directional promoter reporter system would be preferable. Therefore, in the present work, we merge this strategy into an integrative approach for exploring bacterial communities through the lens of their regulatory dynamics, focusing on the study of bacterial promoter elements from environmental soil samples.

Although both constitutive and inducible promoters can be potentially detectable by the bi-directional method, we have focused exclusively on the study of the former, as a proof of concept, avoiding substrate-based induction assays (Uchiyama et al., 2005; Williamson et al., 2005; Uchiyama and Miyazaki, 2010; Guazzaroni et al., 2013). We have collected soil samples from two differentially biomass-enriched sites of a Secondary Atlantic Forest in South-eastern Brazil and generated metagenomic libraries in a bi-directional probe vector for primary screenings. We have characterized the expression behaviors of a large set of GFPlva expressing clones from both libraries and narrowed down our selection to 10 clones for an in-depth analysis regarding potential ORFs and endogenous promoters. By cross-validating *in silico* analyses and experimental data of predicted constitutive promoters, we have located and profiled the expression of 33 endogenous promoters within the selected clones, providing resourceful information concerning the architecture and transcriptional dynamics of promoters from metagenomic fragments. Thought the identification of novel constitutive, natural promoters, our work contributes to the expansion of the toolbox of synthetic biology, which, in turn, can be used for genetic modification of microorganisms relevant in Biotechnology.

## Materials and methods

### Bacterial strains, primers, plasmids, and general growth conditions

*Escherichia coli* DH10B (Invitrogen) cells were used for cloning and experimental procedures. *E. coli* strains were routinely grown at 37°C in Luria-Broth medium or M9 minimal medium (Sambrook et al., 1989) (6.4 g/L Na_2_HPO_4_·7H_2_O, 1.5 g/L KH_2_PO_4_, 0.25 g/L NaCl, and 0.5 g/L NH_4_Cl) supplemented with 2 mM MgSO_4_, 0.1 mM casamino acid, and 1% glycerol as the sole carbon source. When required, chloramphenicol (Cm) (34 μg/mL) was added to the medium to ensure plasmid retention. When cells were grown in minimal medium, antibiotics were used at half concentrations. Transformed bacteria were recovered on LB (Luria–Bertani) liquid medium for 1 h at 37°C and 180 r.p.m, followed by plating on LB-agar plates at 37°C for at least 18 h. All constructions were cloned into the pMR1 bi-directional-reporter vector (Guazzaroni and Silva-Rocha, 2014), which carries mCherry and GFPlva, a short-lived variant of GFP.

### Study site, soil sampling, and DNA extraction

Soil samples were obtained from a parcel of southeast region of Brazil (South America), from a Secondary Atlantic Forest at the University of Sao Paulo (Ribeirão Preto, São Paulo, Brazil; 21°09'58.4”S, 47°51'20.1”W, at an altitude of 540 m). The soil from those parcels are geologically considered Oxisols (Schaefer et al., 2008)—clay soil always presenting a red or yellowish color, due to the high concentration of iron (III) and aluminum oxides and hydroxides—. The top soil from two sections of the parcel (herein referred to as USP1 and USP3) were sampled at a depth of 0–15 cm on July 2015 (soil temperature 23°C). Three replicates (0.2 kg each) were collected within a 1 m distance, and the samples were stored at −20°C until DNA was extracted. Each sample was differentially enriched regarding tree species abundance on plant-litter composition: (i) enriched in leaves from *Phytolacca dioica* and (ii) from *Anadenanthera* spp. DNA was extracted from soil samples using the UltraClean™ Soil DNA isolation Kit (Mo Bio Laboratories, Solana Beach, CA, USA). DNA was visualized by using 0.7% (w/v) agarose gel electrophoresis and quantified spectrophotometrically (260 nm).

### Metagenomic libraries construction and screening for fluorescent clones

For the construction of the libraries, metagenomic DNA was partially digested using Sau3AI, and fragments from 1.5 to 7 kb were extracted from an agarose gel for ligation into the dephosphorylated and BamHI-digested pMR1 vector. Ligation mixtures were transformed by electroporation into *E. coli* DH10B cells. To amplify the libraries, they were grown on LB agar plates containing Cm and incubated for 18 h at 37°C. Both green and red clones were manually isolated from LB-agar plates exposed to blue light wavelength (at ~470 nm) by a transilluminator (Safe Imager™ 2.0 Blue Light Transilluminator). Ten fluorescent and 20 non-fluorescent clones were randomly picked from each library and had their plasmids extracted, following digestion with EcoRI and SmaI enzymes for checking presence/absence of inserts and their sizes. Cells from the same library were collected and pooled together in LB supplemented with 10% (wt/vol) glycerol for storing at −80°C. The plasmids from the 10 selected clones were isolated from individual clones and transformed into new *E. coli* DH10B cells to reconfirm expression patterns.

### Nucleic acid techniques

DNA preparation, digestion with restriction enzymes, analysis by agarose gel electrophoresis, isolation of DNA fragments, ligations, and transformations were done by standard procedures (Sambrook et al., 1989). Plasmid DNA was sequenced on both strands by primer walking using the ABI PRISM Dye Terminator Cycle Sequencing Ready Reaction kit (PerkinElmer) and an ABI PRISM 377 sequencer (Perkin-Elmer) according to the manufacturer's instructions.

### GFP fluorescence assay and data processing

To measure promoter activity, freshly plated single colonies were grown overnight in M9 medium supplemented with required antibiotics. Samples were diluted 1:20 (v/v) in M9 medium for a final volume of 200 μL in 96-well microplates. Cell growth and GFP fluorescence were quantified using a Victor X3 plate reader (PerkinElmer, Waltham, MA, USA). Promoter activities were expressed as the emission of fluorescence at 535 nm upon excitation with 485 nm light and then normalized with the optical density at each point (reported as fluorescence/OD_600_) after background correction. Background signal was evaluated with non-inoculated M9 medium and used as a blank for adjusting the baseline of measurements. *E. coli* DH10B harboring the pMR1 empty plasmid was used as a negative control. Three different positive controls were used, consisting in *E. coli* DH10B harboring pMR1 plasmid with one of the following synthetic constitutive promoters from the iGEM BBa_J23104 Anderson's catalog (http://parts.igem.org/Promoters/Catalog/Anderson) (Kelly et al., 2009) upstream a GFPlva reporter: J23100, J23106, and J23114 (referred here as p100, p106 and p114, respectively; Sanches-Medeiros et al., 2018). Unless otherwise indicated, measurements were taken at 30 min intervals over 8 h. All experiments were performed with both technical and biological replicates, being biological triplicates evaluated as independent measurements on different dates. Raw data were processed and plots were constructed using Microsoft Excel. All data was normalized by background values and transformed to a log2 scale for better data visualization. Heatmap dendrograms with expression profiles were generated by using MeV2 (http://mev.tm4.org/) software.

### Small-DNA inserts libraries generation and screening

In order to experimentally find and validate the promoter regions from each of the 10 selected metagenomic clones, an experimental technique was developed based on the previously described methodology of metagenomic library construction. All selected clones had their plasmids extracted and pooled together in an equimolar ratio. The pooled sample was amplified through a single PCR reaction using high-fidelity polymerase enzyme (Phusion) and previously described primers flanking the MCS region (Multiple Cloning Site) of the pMR1 vector, into which the metagenomic inserts were cloned. The resulting amplicons were firstly submitted to an analytical digestion followed by electrophoretic analysis for finding the optimal concentration of Sau3AI enzyme for obtaining fragments size ranging from 0.1 to 0.5 kb. Then, the purified pooled samples were fragmented by Sau3AI in preparative digestion and thereafter punctured from a 1% agarose gel in the region between 0.1 and 0.5 kb. These small DNA fragments, in turn, were ligated to pMR1 vector. Aliquots of electrocompetent *E. coli* DH10B cells were transformed with ligated DNA. A total of 100 fluorescent clones (80 expressing GFP and 20 expressing mCherry) were isolated under blue light excitation screening and had their plasmids extracted for sequencing reactions. Fluorescent clones were stored at −80°C in LB medium supplemented with required antibiotics and 10% glycerol (v/v).

### *In silico* analysis of ORFs and promoter regions

The inserts of selected clones were sequenced on both strands as previously described. Sequences were manually assembled for the generation of 10 contigs. All sequences were analyzed for taxonomic origins by using the *PhylopythiaS* Web Server (Patil et al., 2012) (http://phylopythias.bifo.helmholtz-hzi.de/index.php?phase=wait), a sequence composition-based classifier that utilizes the hierarchical relationships between clades. Putative ORFs were identified and analyzed using the online ORF Finder platform, available at the NCBI website (https://www.ncbi.nlm.nih.gov/orffinder/). Comparisons of nucleotide and transcribed amino acid sequences were performed against public databases (NCBI) using BlastN, BlastX, and BlastP (BLAST, basic local alignment search tool) at the NCBI on-line server. For translation to protein sequences, the bacterial code was selected, allowing ATG, GTG, and TTG as alternative start codons. All the predicted ORFs longer than 270 bp were translated and used as queries in BlastP. Sequences with significant matches were further analyzed with psiBlast, and their putative function was annotated based on their similarities to sequences in the COG (Clusters of Orthologous Groups) and Pfam (Protein Families) databases. Predicted general cellular functions were annotated only for known ORFs based on the MultiFun classification (Serres and Riley, 2000). All sequences with an E-value higher than 0.001 in the BlastP searches and longer than 300 bp were considered to be unknown. Transmembrane helices were predicted with TMprep (http://www.ch.embnet.org/software/TMPRED_form.html) and signal peptides with Signal P3.0 server (http://www.cbs. dtu.dk/services/SignalP/). A complete table can be found at Table S1. Promoter prediction was based on the analysis of the 10 contigs by using both BPROM (http://www.softberry.com/berry.phtml?topic=bprom&group=programs&subgroup=gfindb) and bTSSfinder (http://www.cbrc.kaust.edu.sa/btssfinder/) web-based platforms. Both methods searched for rpoD-related sequences and we have only considered as valid predictions the ones matched on both approaches. Those filtered sequences were used to cross-validate 23 out of 33 experimentally defined regulatory regions by comparing the positions between predicted and experimental sequences in metagenomic fragments. The positions of the 33 small DNA fragments were obtained by a multiple alignment of the original contigs (queries) against those selected sequences, which has also allowed the validation of the promoter's directionality—forward or reverse—by observing the matched strands (Plus/Plus or Plus/Minus). The consensus Logo sequence was based on the alignment of the 33 experimentally validated promoters, using the WebLogo platform (http://weblogo.berkeley.edu/logo.cgi).

### Criteria for the choice of sample sizes

The sample sizes chosen in this work were based on a seminal study regarding the characterization of random promoter libraries (Cox and Elowitz, 2007) in which ~1% (288) of the total set of promoters (22,000) was selected for further analysis. In our study, we have selected a much higher fraction of the population for sampling (~25% of 1,100 screened clones). Furthermore, using classical statistics for determining optimal sample sizes and reducing the uncertainty caused by sampling error (Nakagawa and Cuthill, 2007), we have found that sampling 260 clones from a total of 1,100 clones would result in confidence level of 99% with a confidence interval of 0.07. Each selected clone was manually streaked in LB-agar and microbiologically purified two times for further validation in plate reader assays—which was done with biological and technical triplicates. Regarding the 10 selected clones at the in-depth analysis, we have adopted the same sample fraction from the study of (Cox and Elowitz, 2007), (1% of the total number of positive clones-−10 in 1,100 clones). In this context, from each of the 10 analyzed clones containing metagenomic fragments we have obtained at least three promoters, which were individually characterized in plate reader assays. The choice of 100 clones from the small-fragment library was based on the following rationale: (i) the combined size of the 10 selected clones in this analysis was 30 kb, (ii) each small fragment had an average of 0.4 kb, thus, (iii) 100 fluorescent clones from the small-insert library would represent ~40 kb, providing enough coverage for all 10 original clones. Furthermore, as each fluorescent clone would represent a single promoter sequence at a specific region in the original clones, it was highly improbable that the 100 selected clones would cover the 10 original clones. Thus, our intention by choosing a sample size of 100 clones was to enrich the single promoters. This assumption was further supported by the discovery of only 33 promoters among those 100 sequences (promoter sequences were overrepresented).

## Results

### Generating metagenomic libraries and screening for fluorescent clones

**Figure 1:**
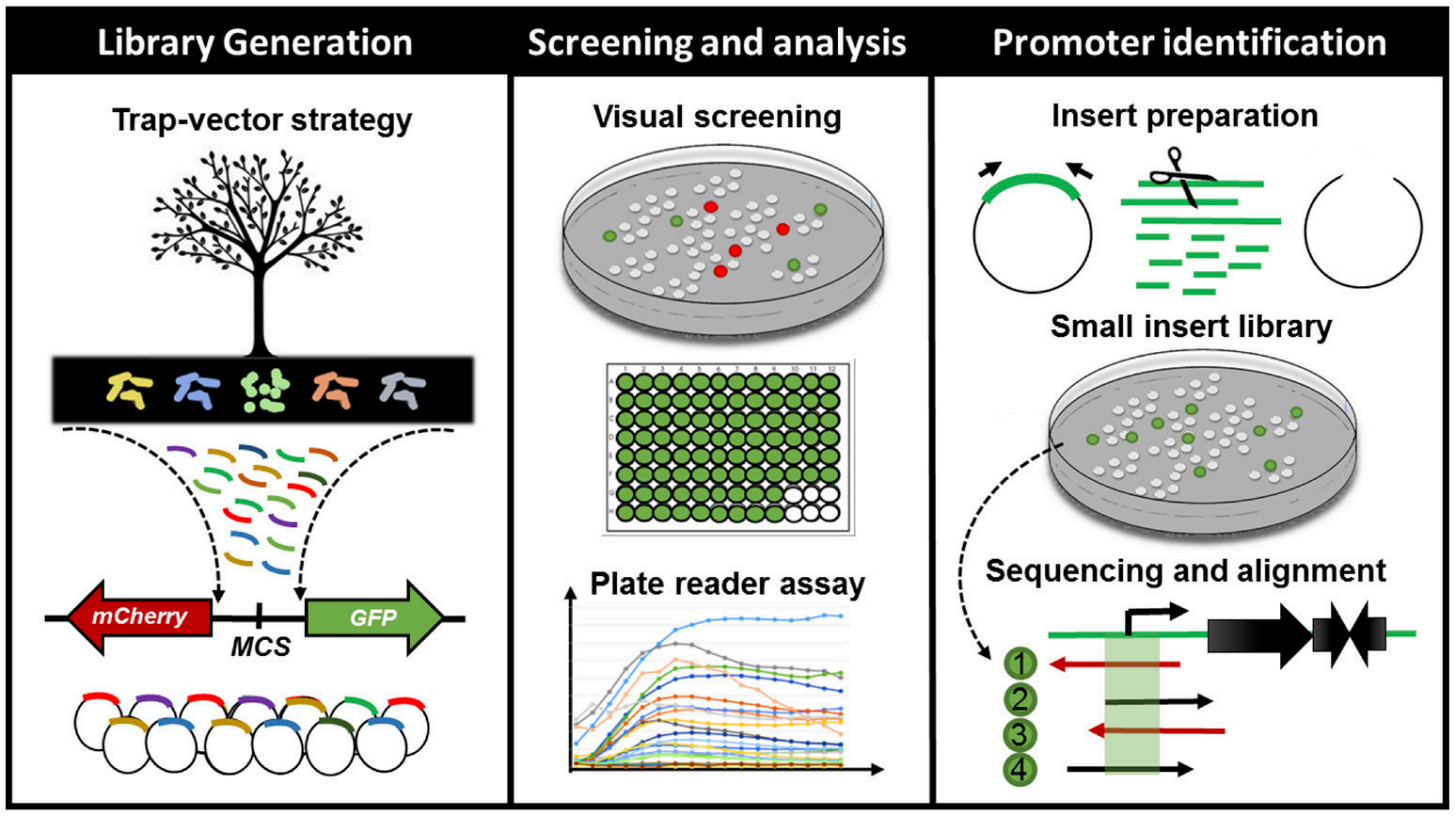
Schematic representation of the workflow for finding, characterizing and cross-validating novel bacterial cis-regulatory elements in environmental samples. From left to right: firstly, we have generated metagenomic libraries from soil samples in *E. coli* DH10B. The DNA fragments were cloned into a bi-directional reporter trap-vector (bearing *mCherry* and *GFPlva* fluorescent reporters), pMR1, which allowed for the screening of promoters in both DNA strands. Secondly, we have manually screened all visible fluorescent clones from our metagenomic libraries and analyzed the expression patterns of all green fluorescent clones on a microplate reader during 8 h. Lastly, we have selected 10 clones based on their GFPlva expression patterns for an in-depth analysis combining experimental (small DNA insert library generation) and *in silico* promoter prediction. This integrated strategy has allowed us to identify, validate and estimate the accessibility of novel promoter regions from metagenomic libraries.

We have constructed and assessed two metagenomic libraries hosted in *E. coli* DH10B strain for the analysis of bacterial promoters in environmental samples (Figure 1). The libraries were generated from soil microbial communities of two sites bearing differential tree litter composition (*Anadenanthera* spp. and *Phytolacca dioica*) within a Secondary semi-deciduous Atlantic Forest zone at the University of Sao Paulo, Ribeirão Preto, Brazil—see Experimental Procedures for further details. Both metagenomic DNA were cloned into the pMR1 (Guazzaroni and Silva-Rocha, 2014) bi-directional reporter vector—which has a *GFPlva* and a *mCherry* reporter gene in opposite directions, flanking a multiple cloning site; chloramphenicol resistance marker and a *p15a* origin of replication for low/medium copy number. Each metagenomic library presented about 250 Mb of environmental DNA distributed into ~60,000 clones harboring insert fragments size ranging from 1.5 to 7 kb, with an average size of 4.1 kb (Table 1). We have chosen fragments of 1.5–7 kb in order to validate our strategy on standard-sized functional metagenomic libraries based on plasmid vectors (Gabor et al., 2004; Uchiyama et al., 2005; Pushpam et al., 2011; Jiménez et al., 2012; Guazzaroni et al., 2013). In total, 1,100 fluorescent clones, resulting in a rate of approximately one fluorescent clone every 150 clones (USP1) or every 90 clones screened (USP3), were manually selected under blue light exposition. Then, these fluorescent clones were directly recovered from LB agar plates supplemented with chloramphenicol. The direct screening was preferred over the use of metagenomic clone pools from stocks as it reduces the chances of both biased clone enrichment (e.g., clones with higher growth rates, usually clones bearing small inserts or without insert) and dilution of positive clones with impaired growth (e.g., clones with high expression of GFP and/or other exogenous genes), avoiding thus clonal amplification.

**Table 1:** Features of the generated metagenomic libraries.

**Metagenomic library**	**USP 1**	**USP 3**
Total number of clones	100,000	90,000
Percentage of clones with insert (%)	60	70
Number of clones with insert	60,000	63,000
Total number and rate^*^ of fluorescent clones	400 (1:150)	700 (1:90)
Total number and rate^*^ of green clones	270 (1:220)	400 (1:157)
Total number and rate^*^ of red clones	130 (1:460)	300 (1:210)
Average insert size (kb)	4.5	3.7
Total metagenomic library size (Mb)	270	233
Estimated number of genomes^**^	60	52

* *Rate represented by the number of fluorescent clones divided by the total number of clones with inserts*.

** *Assuming 4.5 Mb per genome (Raes et al., 2007)*.

### Evaluating the expression dynamics of fluorescent clones

**Figure 2:**
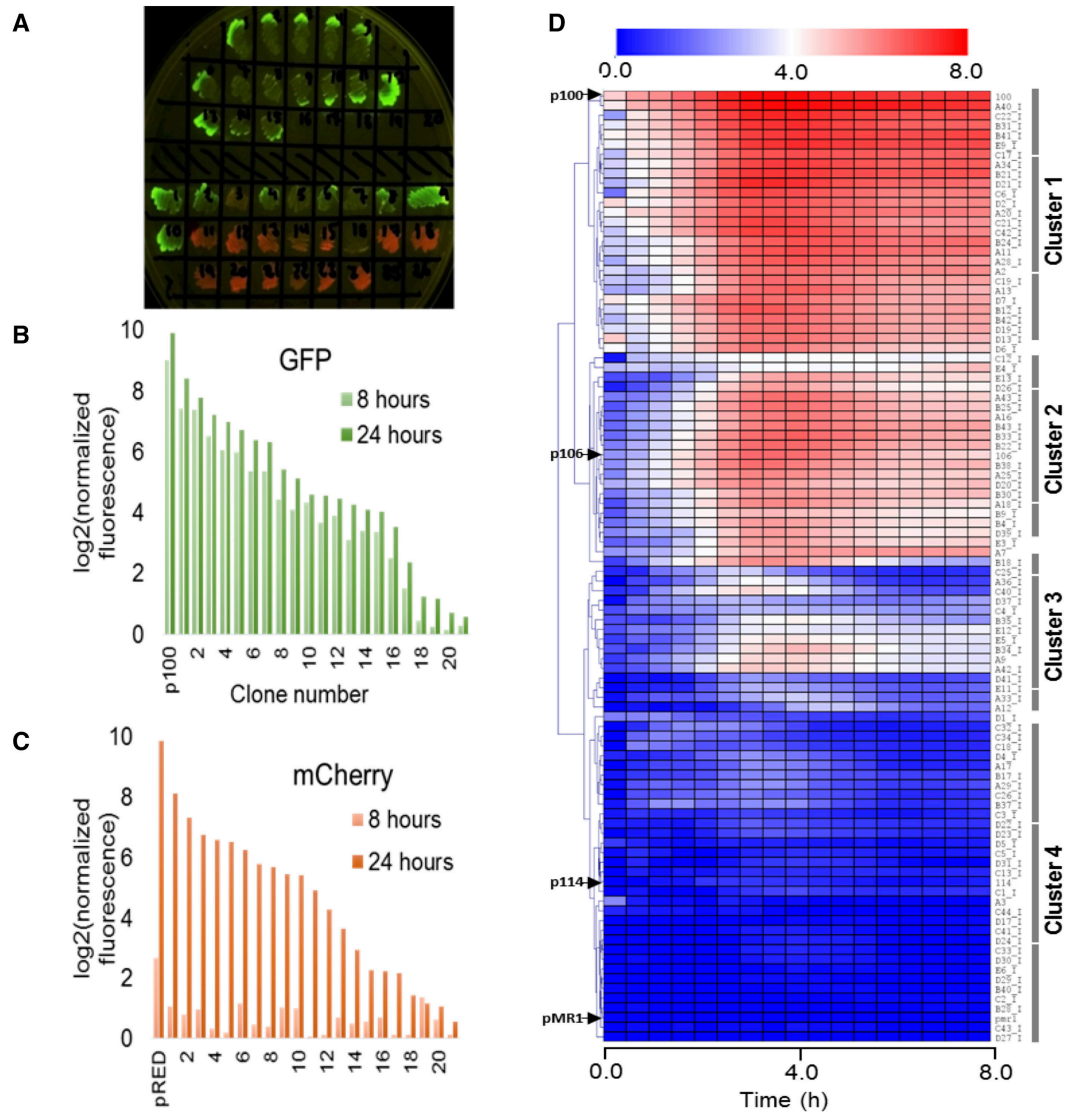
Evaluating the expression dynamics of fluorescent clones. **(A)** LB-agar plate under blue light excitation comprising a subset of metagenomic isolated clones expressing GFPlva (top) and mCherry (bottom) fluorescent reporters. A few clones were observed to express both reporters. All isolated clones were initially considered to hold at least one endogenous promoter. **(B,C)** Indirect assessment of maturation times from both fluorescent reporters GFPlva **(B)** and mCherry **(C)** after 8 h (light bars) and 24 h (dark bars) of the beginning of the experiment. Maturation times are substantially lower for mCherry than for GFPlva, which excluded the former from further analyses. Positive controls for GFP and mCherry are represented by p100 and pRED, respectively. Fluorescence data has been normalized by OD_600_ values for each sample following normalization by values from the negative control (empty-pMR1). Data was transformed to log2 scale to allow better visualization of fluorescence variation. **(D)** Hierarchical representation of a metaconstitutome (i.e., all expression profiles from a single metagenomic library (USP3) in *E. coli*. Fluorescence time-lapse dynamics were measured during 8 h for each clone and represented as heat maps. Promoter activities (calculated as GFP/OD_600_) were normalized by the negative control (*E. coli* DH10B harboring empty pMR1) and transformed to log2 scale in order to facilitate the visualization of subtle activities. Positive controls (p100, p106, and p114-strong, medium and low expression, respectively) and negative control (pMR1) expression profiles are indicated by black arrows at the left side of the heatmap. Data are representative of three independent experiments.

In order to analyse the expression patterns of the isolated clones, we evaluated the intrinsic dynamics of GFPlva and mCherry by randomly selecting 20 clones expressing each reporter (as schematically represented in Figures 1, 2A). As represented in Figures 2B,C, we found that clones expressing mCherry were not suitable for standard microplate 8 h assays, as the fluorescence intensity values differed dramatically between 8 and 24 h after the beginning of the experiment. The slow kinetics of mCherry expression has already been reported as a consequence of a two-step oxidation process for protein maturation when compared to the one-step maturation process found in GFP reporters (Hebisch et al., 2013). We highlight that although mCherry clones were not optimized for dynamic profiling, they were essential for quantifying the total number of metagenomic fragments harboring promoters accessible to *E. coli*–the sum of both green and red fluorescent clones in the library. On the other hand, the clones expressing GFPlva presented the enhanced intrinsic properties for microplate assays, supported by the observation of very similar fluorescence intensities between the two time points tested. Furthermore, the GFPlva has an LVA-degradation tag attached to its C-terminal, which reduces GFP accumulation and increases protein turnover, generating a more precise fluorescence output on analysis of expression patterns (Andersen et al., 1998).

Thus, 260 clones expressing GFPlva—see Experimental Procedures for further information about chosen sample sizes—(160 clones from the USP1 library and 100 from USP3) were selected for further analysis of expression patterns on microplate reader assays with biological and technical triplicates. The dynamic profiles for each clone were converted into heat maps and hierarchically clustered by a Euclidean Distance algorithm into a dendrogram, concisely representing the expression patterns of each metagenomic library. In order to assess the diversity of promoter strengths among the generated metagenomics libraries, three previously characterized constitutive promoters (see Experimental Procedures for further information) positioned upstream a GFPlva reporter were used as standards for strong, medium and weak expression profiles (referred here as p100, p106, and p114, respectively).

Considering both metagenomics libraries, we have found a total of 30 strong promoters showing a strength similar to the p100 control, 40 medium strength promoters similar to the p106 control, 60 weak promoters similar to the p114 control and a wide range of promoters with particular expression patterns which did not cluster with any of the previously mentioned positive controls (Figure 2D and Figure S1). Moreover, the dynamic expression profiles have allowed us to observe a few clones that, although constitutively active, had their GFPlva expression levels increased during certain time frames (Figure 2D). Concerning the hierarchical organization of the expression profiles, the dendrogram of the USP3 library (Figure 2D) could be subdivided into at least four well-defined expression clusters comprising: (i) high, (ii) medium, (iii) low and (iv) very low expression profiles. A very similar pattern was identified in the expression dendrogram independently generated for the USP1 metagenomic library (see Figure S1).

### *In silico* analysis of DNA metagenomic fragments from selected clones

From the 260 assessed samples, we have selected 10 clones displaying particular profiles (see Figure S2)—see Experimental Procedures for further information about chosen sample sizes—depicting the diversity of expression behaviors found in both libraries. The inserts from selected clones were sequenced and analyzed for C-G content, taxonomic origins, potential ORFs and RpoD-related promoter regions (−10 and −35 conserved regions). The relative abundance of the guanine-cytosine content of each insert was assessed (Table 2), resulting in a median of 54%, varying from 43 to 61%, indicating their diverse phylogenetic affiliation. Using the *PhylopythiaS* sequence classifier for metagenomic sequences (Koonin, 2009; Patil et al., 2012), the DNA fragments were assigned to their closely related phylum (Table 2 and Figure S3). The most abundant assigned phyla were Proteobacteria (46%), followed by Actinobacteria (23%), Verrumicrobia (15%), Chloroflexi (8%) and Bacteroidetes (8%) (see Figure S3).

**Table 2:** Description of the ORFs contained in plasmids from the selected clones (pCAW1 to pCAW10) and their sequence similarities.

**Clone_Sample [insert bp]**	**G + C %**	**GenBank accession No**.	**Phylum^a^**	**ORF^b^**	**Strand**	**Length (aa^c^)**	**Closest similar protein^d^ (Length in aa)**	**Closest Organism/Phylum^e^**	**Identity (%)**	**Putative function**
**pCAW1 (2,367 bp)**	55%	KY939589	Proteobacteria or Verrucomicrobia	1	Minus	131	Hypothetical protein (416)	*Bacteriodetes bacterium/Proteobacteria*	68%	Alginate lyase
				2	Plus	271	Hypothetical protein (261)	*Acidobacteria bacterium/Acidobacteria*	73%	17-B-hydroxysteroid dehydrogenase
				3^b^	Plus	295	Beta-glucosidase (777)	*Caulobacter* sp. *OV484/Proteobacteria*	66%	Beta-glucosidase
**pCAW2 (2,069 bp)**	52%	KY939590	Actinobacteria	1	Plus	304	Unkonwn^c^	*Hyphomicrobium* sp. *NDB2Meth4/Proteobacteria*	33%	Unknown
				2	Plus	249	Unkonwn	*Hungatella hathewayi/Firmicutes*	33%	Unknown
**pCAW3 (4,404 bp)**	53%	KY939591	Proteobacteria	1	Minus	318	IS4 family Transposase (320)	*Escherichia coli/Proteobacteria*	96%	IS4 family transposase
				2	Minus	1011	DNA-directed RNA polymerase subunit beta' (1430)	*Sphingobacteriales bacterium 44-61/Bacteroidetes*	83%	RNA polymerase - Beta Subunit
				3	Plus	120	Uncharacterised protein (135)	*Bordetella pertussis/Proteobacteria*	47%	Unknown
				4	Plus	151	Uncharacterised protein (130)	*Bordetella pertussis/Proteobacteria*	37%	Unknown
				5	Plus	94	Uncharacterised protein (64)	*Bordetella pertussis/Proteobacteria*	82%	Unknown
				6	Plus	96	Uncharacterised protein (86)	*Vibrio cholerae/Proteobacteria*	48%	Unknown
				7	Plus	173	predicted protein (585)	*Ruminococcus* sp. *CAG:403/Proteobacteria*	26%	Unknown
**pCAW4 (4,002 bp)**	61%	KY939592	Proteobacteria	1	Minus	245	Nosine monophosphate cyclohydrolase (246)	*Ktedonobacter racemifer/Chloroflexi*	63%	IMP cyclohydrolase
				2	Minus	214	Phosphodiesterase (498)	*Candidate division NC10 bacterium/NC10*	40%	Phosphodiesterase
				3	Minus	402	Hypothetical protein A2Y08_02680 (625)	*Planctomycetes bacterium GWA2_40_7/Planctomycetes*	43%	Unknown
				4^b^	Plus	142	Gentisate 1,2-dioxygenase (349)	*Pseudomonas* sp. *21C1/Proteobacteria*	60%	Gentisate 1,2-dioxygenase
**pCAW5 (2,724 bp)**	54%	KY939593	Verrucomicrobia	1^b^	Plus	642	Pyruvate:ferredoxin oxidoreductase (1565)	*Uncultured bacterium HF770_11D24]/Acidobacterium*	80%	Pyruvate:ferredoxin oxidoreductase
**pCAW6 (2,125 bp)**	57%	KY939594	Chloroflexi or Proteobacteria	1	Plus	159	Hypothetical protein BGO39_33875 (215)	*Chloroflexi bacterium 54-19/Chloroflexi*	65%	MerR family
				2	Plus	336	Hypothetical protein BGO39_33870 (347)	*Chloroflexi bacterium 54-19/Chloroflexi*	78%	PrsW intramembrane metalloprotease
				3^b^	Plus	163	Hypothetical protein BGO39_33865 (173)	*Chloroflexi bacterium 54-19/Chloroflexi*	75%	Chromate transporter
**pCAW7 (2,558 bp)**	46%	KY939595	Actinobacteria	1^b^	Minus	391	Hypothetical protein A2X07_06330 (480)	*Flavobacteria bacterium GWF1_32_7/Bacteroidetes*	45%	Por secretion system sorting domain
				2	Minus	250	Hypothetical protein (586)	*Chitinophagaceae bacterium PMP191F/Bacteroidetes*	65%	Polysaccharide Lyase
**pCAW8 (4,480 bp)**	57%	KY939596	Actinobacteria	1	Plus	508	Hypothetical protein AUH20_02325 (597)	*Rokubacteria bacterium/Rokubacteria*	76%	5-oxoprolinase / Hydantoinase_B
				2	Minus	348	Oxidoreductase (336)	*Rokubacteria bacterium/Rokubacteria*	61%	Flavin-utilizing monoxygenases
				3	Plus	314	Hypothetical protein ETSY1_46935 (279)	*Candidatus Entotheonella* sp. *TSY1/Tectomicrobia*	76%	Cellulose biosynthesis BcsQ
**pCAW9 (2,573 bp)**	43%	KY939597	Bacteroidetes or Proteobacteria	1^b^	Minus	81	Hypothetical protein (129)	*Janthinobacterium/Proteobacteria*	50%	Unknown
				2	Minus	303	Formylglycine-generating enzyme (379)	*Mucilaginibacter* sp.*/Bacteroidetes*	65%	Formylglycine-generating enzyme
				3	Minus	457	Acetylglucosamine-6-sulfatase (504)	*Flavihumibacter solisilvae/Bacteroidetes*	67%	Acetylglucosamine-6-sulfatase
**pCAW10 (2,076 bp)**	56%	KY939598	Proteobacteria	1	Plus	204	Hypothetical protein (195)	*Luminiphilus syltensis/Proteobacteria*	50%	Unknown

a Classification based on PhylopythiaS (Patil et al., 2012) webserver

b Truncated proteins

c aa, amino acids

d Sequences with an E-value higher than 0.001 in Blastp searches were considered to be unknown proteins

e *Classification based on Blastp*.

In the case of the identification of putative genes, 29 ORFs with significant *E-values* (<0.001) were found (Table 2) unevenly distributed between both DNA strands, in line with a lack of strong directional trends regarding bacterial genome organization (Koonin, 2009). The ORFs were also classified within a range of functional classes (delineated by MultiFun; Serres and Riley, 2000) and taxonomic groups based on closest similar proteins (Table 2). Regarding gene function, the most abundant ORFs were related to unknown functions (31%) and metabolism (31%), followed by stress adaptation cell processes (17%) (Table 2).

The *in silico* promoter prediction has also provided relevant information concerning the potential number of regulatory regions on each selected fragment. The BPROM software (Solovyev, 2011) has been extensively employed in other promoter prediction studies and is based on the analysis of the −35 and −10 consensus sequence of RpoD promoters. The main sigma subunit, sigma-70 encoded by *rpoD*, plays a major role in transcription of growth-related genes, the so-called housekeeping genes (Lonetto et al., 1992; Gruber and Gross, 2003; Paget and Helmann, 2003). From the *in silico* analysis, a total of 140 promoters were predicted among the 10 selected clones, suggesting an average of 5 RpoD-related promoters/kb. This led us to question whether most expression profiles previously described (Figure 2D and Figure S1) were representing the dynamics of a single “dominant” promoter or the combined effect of multiple adjacent promoters present in the metagenomic fragment. Considering that, we have delineated a strategy to experimentally assess the number and location of accessible promoters from our selected clones, contrasting experimental results with *in silico* data.

### Experimental identification, characterization, and cross-validation of promoter regions

**Figure 3:**
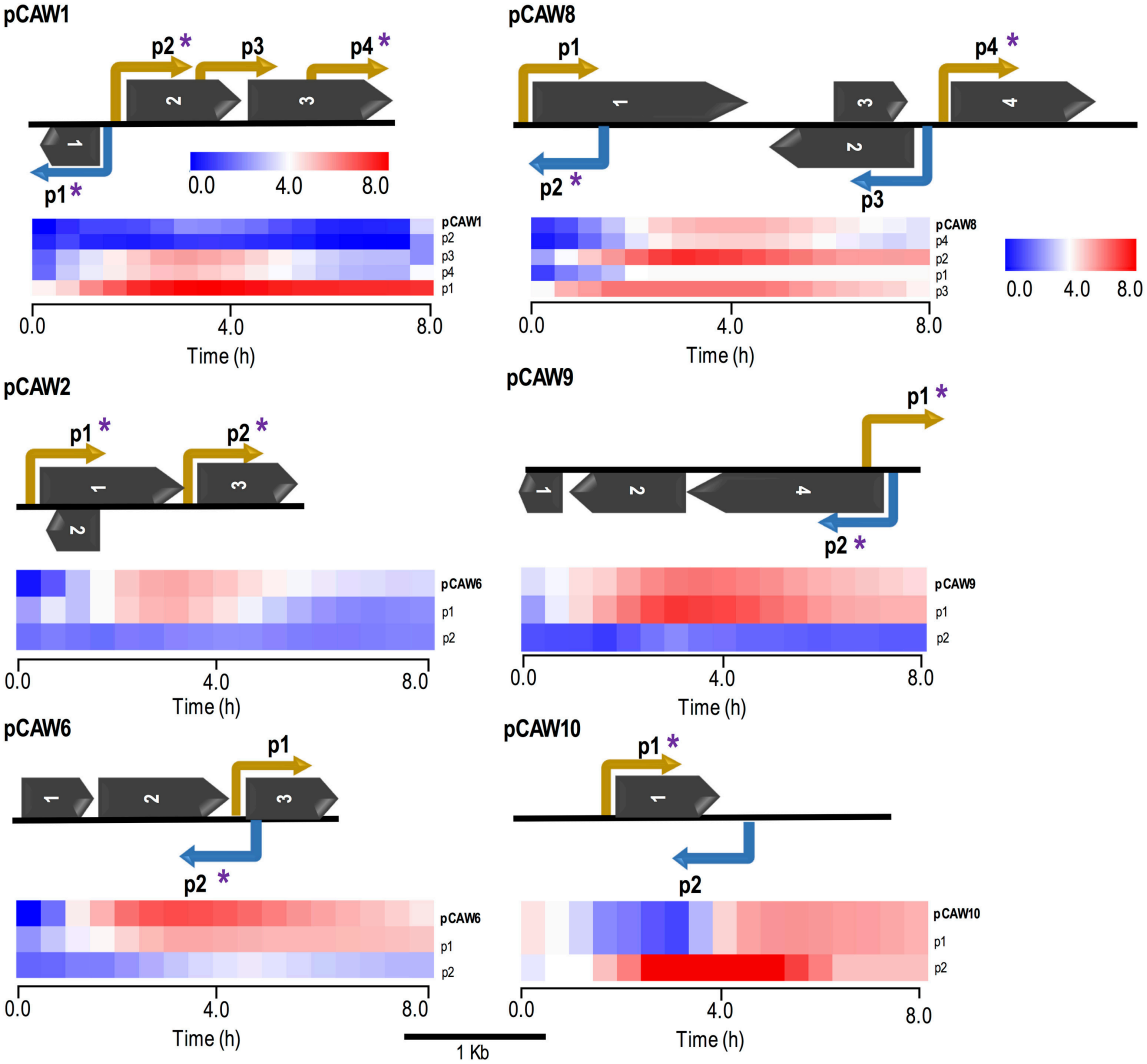
Schematic representation of six metagenomic inserts (contigs) showing predicted ORFs and experimentally validated/characterized promoters. Each contig is identified on the far left of each subfigure. Promoters are indicated by elbow-shaped arrows and name according to their relative position in the contig. Promoter directionality, regarding the leading and lagging strands, is represented by yellow and blue colors, respectively. Asterisks over specific promoters indicate regulatory regions which were cross-validated by matching *in silico* predictions. Dark arrows represent predicted ORFs, according to their relative positions in each contig (see Table 2 for more information). All genetic features respect their original relative sizes, following the 1 kb scale depicted at the bottom of this figure. Beneath each metagenomic insert, there is a heat map cluster representing the whole set of promoter activities measured during 8-h fluorescence assays. The first line of each cluster shows the original expression profile initially measured for each metagenomic insert. All other lines represent expression activities from *de novo* experimentally validated promoters within each contig (small DNA fragments). The second line of each cluster represents the endogenous promoter showing the most similar activity with respect to the original expression profile for each contig. All expression profiles are properly identified at the most rightmost side of each line, following their respective contig/promoter name. For the supplementary set of analyzed contigs, see Figure S4.

In order to explore the potential set of accessible promoter regions from our metagenomic libraries, we developed a small DNA insert library generation approach (Figure 1). Firstly, the plasmids from the previously 10 selected clones (original clones) were pooled together for insert amplification in a single PCR reaction. The resulting amplicons were fragmented by Sau3AI digestion and DNA fragments ranging from 0.1 to 0.5 kb were selected for subsequent cloning into the pMR1 vector. The generation of this sub-fragment library allowed the screening for both red and green fluorescent colonies as they would represent the accessible set of promoters among the metagenomic DNA fragments studied. It is important to highlight that as the cloning process was not directed, small fragments bearing promoter regions had a 50% chance of getting cloned in any direction, thus clones expressing mCherry were also isolated for subsequent sequencing. A total of 100 clones—see Experimental Procedures for further information about chosen sample sizes—coming from the small DNA insert library (80 expressing GFPlva and 20 expressing mCherry) were sequenced and then aligned against the original metagenomic fragments. As a result, we have identified at least 33 promoter regions within the initial set of the selected metagenomic clones (Figure 3, Figure S4, and Table S1).

Additionally, the current experimental approach allowed us not only to identify novel promoter regions but also to determine promoter directionality. The evaluation of promoter localization within the 10 selected clones revealed that from the 33 experimentally selected small fragments, 7 (21%) were considered intragenic promoters while the remaining 79% (26 promoters) were considered primary promoters, defined as the furthest upstream promoter in a gene/operon (Conway et al., 2014). For the sake of comparison, *E. coli* K-12 genome presents the following proportions: primary (66.3%), secondary (19.6%), intragenic (9.8%), and antisense (4.2%) promoters (Cho et al., 2009; Conway et al., 2014).

Based on the alignment results, we selected a defined set of small fragment clones related to each original sequence for dynamic expression profiling on a microplate reader. The results showed that for each set of small-fragments belonging to a DNA metagenomic clone, there was at least one with an expression pattern corresponding to the original clone previously observed (Figure 3 and Figure S4). Similarly, we identified other clones bearing small-inserts with individual profiles different to the primarily observed, representing alternative promoter regions in the original sequence that were not mapped in the initial approach (Figure 3). Data has also shown that, in our experimental conditions, it seems that in each case a single promoter (usually the closest to the reporter gene) has the major contribution for the gene expression pattern observed. This can be concluded since, in each case, only one promoter mapped from the small-insert library produced the same expression profile observed for the original full length fragment.

**Figure 4:**
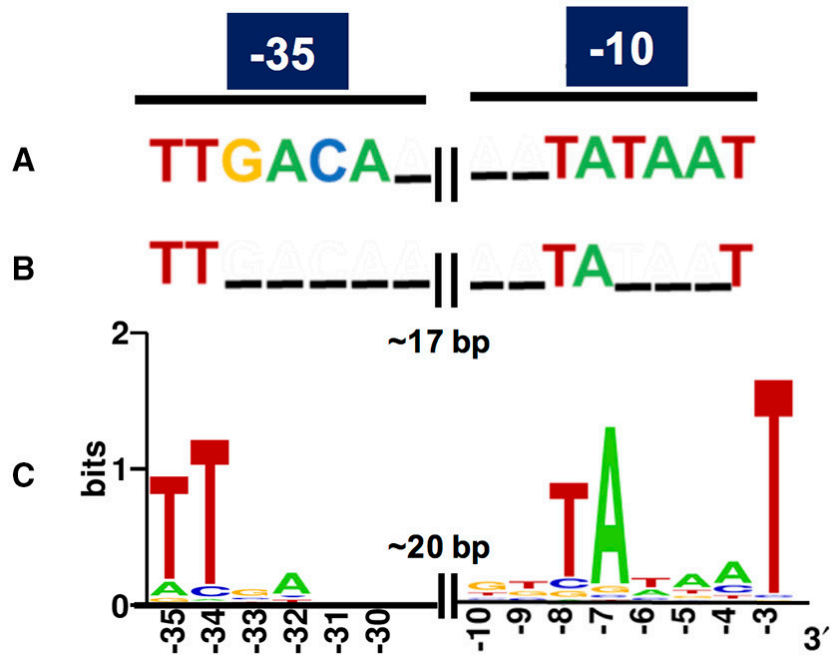
Consensus of RpoD-related metagenomic promoters. **(A)** Known consensus sequences of the RpoD-dependent promoter determined *in vitro*, TTGACA (−35) and TATAAT (−10) separated by 17 plus/minus 2 bp in *E. coli* (Shimada et al., 2014). **(B)** Known consensus sequences of 582 promoters experimentally validated in *E. coli* (Shimada et al., 2014; Gama-Castro et al., 2016; Keseler et al., 2017). **(C)** The sequences of the 33 promoters experimentally validated in this study were aligned and subjected to Logo analysis (Crooks et al., 2004). The consensus from the metagenomic set **(C)** is very similar to the one from the experimentally validated set from *E. coli* **(B)**.

Regarding *in silico* cross-validation, from the 33 experimentally validated promoters, 23 RpoD-related promoters (70%) were supported by the algorithmic analysis as they were aligned to their respective original sequences (Figure 3). On the other hand, the remaining 10 sequences (30%) were considered as promoters exclusively identified by experimental approaches. This could indicate that these promoters that do not macth the RpoD concensus are reconigzed by alternative sigma factors. This hypothesis will be investigated in future studies. Finally, sequences of the above experimentally validated promoters were characterized accordingly to previous studies reported in the literature. For this, we adopted an *in silico* classification proposed by Shimada et al. (2014) (Shimada et al., 2014), in which constitutive promoters present a high-level conservation of the consensus sequence for the major sigma factor RpoD, that is, the elements TTGACA (−35) and TATAAT (−10) separated by ~17 bp (Figures 4A,B). Constitutive promoters are defined as promoters active *in vivo* in all circumstances, and, on the other hand, inducible promoters are switched ON and OFF by transcription factors depending on the *in vivo* conditions (Shimada et al., 2014). The Logo pattern (Crooks et al., 2004) generated from the alignment of the 33 identified metagenomic promoters (Figure 4C) indicated that positions −35 and −34 (−35 box) and positions −8, −7, and −3 (−10 box) were highly conserved. Additionally, when the promoters were analyzed in sub-groups based on the level of strength (high, medium and low), we could notice a variation in the consensus sequence obtained for each group (Figure S5). These variances in the consensus sequences could explain the different promoter expression profiles observed experimentally.

## Discussion

### Meta-expression profiles for studying microbial communities

The similar expression clusters found between the two independent metagenomic libraries might suggest broader trends of organizational expression patterns in nature. Independent studies on microbial communities from aquatic environments have described similar patterns by evaluating gene expression through metatranscriptomic analysis (Frias-Lopez et al., 2008; Stewart et al., 2012; Dupont et al., 2015; Fortunato and Crump, 2015), indicating that our observations are not restricted to the assessed soil samples. It has also been computationally demonstrated by Fernandez et al. (2014) that the microbial metaregulome—the whole set of regulons of an environmental sample—is shaped by the physicochemical conditions of the environment as an adaptive process. Thus, we suggest that expression profiling of an environmental sample might bear great potential for revealing insightful trends regarding the transcriptional diversity of microbial communities and for aiding on the design of efficient microbial communities for therapeutic or ecological needs (Fernandez et al., 2014; Fredrickson, 2015; Solé, 2015; Johns et al., 2016).

Regarding the explanation for the diversity expression profiles found among the metagenomic clones, it is important to stress that regulatory patterns have a multifactorial nature, being ruled by many different processes. Firstly, the regulatory dynamic is inherently interconnected with the function of the original regulated gene (e.g., housekeeping, adaptive etc.) (Silander et al., 2012). Secondly, the transcriptional bias imposed by the *E. coli* molecular machinery might constraint the recognition of promoter elements and/or not necessarily reproduce the original behaviors found in natural hosts (Gabor et al., 2004; Liebl et al., 2014; Guazzaroni et al., 2015). Another point to be taken into consideration is that artificial juxtaposition of the exogenous promoter to the ribosome-binding site of the fluorescent reporter might increase expression as a consequence of the cloning process. Finally, another process that could influence the detection of active clones in *E. coli* is that the expression of many heterologous genes are toxic to this host (Kimelman et al., 2012). This would also limit the cloning of some fragments in this host for functional metagenomics approaches.

Our observations also suggested transcriptional regulation beyond the control of the RpoD sigma factor for those clones (i.e., adjacent transcription factors), introducing novel niches for the exploration of regulated promoters. Since the discovery of distinct expression behaviors is essential for expanding the current set of commercial promoters, the diversity of expression profiles highlighted in this study has supported the current framework as a promising strategy for finding novel promoters for downstream applications. We also believe the developed strategy could greatly benefit from the combination with other high-throughput screening methods, such as SIGEX (Uchiyama et al., 2005), providing innovative possibilities for the prospection of both inducible and constitutive promoters. Finally, we emphasize our observations are always constrained, to a certain extent, by the perspective of the chosen microbial host (Neufeld et al., 2006; Guazzaroni et al., 2015; Alves Ld et al., 2017) (i.e., the set of constitutive promoters active in *E. coli*) and might represent only a fraction of the effective environmental metaconstitutome. Future studies systematically applying our methodology to a range of environmental samples and hosts will greatly contribute to understanding this relationship between regulatory diversity and environmental adaptation in bacteria.

### Regulatory architectures and host compatibility for promoter exploration

Through the generation of a small-DNA insert library combined to *in silico* platforms we were able to analyse taxonomic and architectural features of the metagenomic fragments. We have also provided both (i) a consensus of recognizable exogenous constitutive promoters in an *E. coli* host. The analysis of the metagenomic fragments for nucleotide composition were in agreement with previous G-C content diversity analyses of soil samples, which ranged from 50 to 61% (Foerstner et al., 2005; Bohlin et al., 2010; Mann and Chen, 2010), suggesting the environmental influence on G-C content and taxonomic predominance of microbiomes. Although phylogenetic affiliation based on ORFs at the protein level are not suitable as sequence-composition based classifiers—as *PhylopythiaS*—for predicting taxonomic origins, we could observe that there was an agreement between both methods in a few samples (e.g., pCAW3, pCAW6, pCAW9 and pCAW10). Furthermore, the abundance of bacterial groups and gene functions predicted in this work was also similar to previous high-throughput studies in soil microbial communities (Janssen, 2006; Fierer et al., 2007, 2012). Considering the above, the proposed experimental methodology has allowed us to directly asses the different bacterial groups that had promoters sequence recognizable by the host–as the metagenomic fragments from these predicted taxa have allowed GFP expression in *E. coli*.

Regarding the in-depth search for promoters *in vivo*—small-DNA library—and *in silico*, the experimental finding of at least 33 promoter regions within the initial set of the selected metagenomic clones suggested the *in silico* prediction was overestimated (140 RpoD-related promoters). The above can be explained since it is not uncommon for prediction algorithms to underestimate or overestimate results due to a lack of information regarding diversity and variability of natural *cis*-regulatory sequences (Vanet et al., 1999; de Jong et al., 2012; Shahmuradov et al., 2016). Furthermore, the analysis of the metagenomic promoter positions/architectures have slightly diverged from the *E. coli* K-12 genome, suggesting the diversity of genomic architectures in metagenomic libraries and a current underestimation of bacterial intragenic promoters that goes far above the *E. coli* model.

Regarding the promoter consensus obtained from the small-DNA fragments, we hypothesized that these sequences could be either recognized by other sigma factors than RpoD or presented unusual consensus sequences for −10 and −35 boxes which have bypassed the algorithmic analysis. However, experimental validation in *E. coli* strains lacking diverse sigma factors genes should be necessary for a more accurate conclusion. Although the observed logo pattern was distant from the *E. coli* consensus proposed for the RpoD-dependent constitutive promoters identified *in vitro* (Figure 4A; Shimada et al., 2014), it was very similar to the previously described consensus from experimentally validated promoter (Mitchell, 2003) sets from RegulonDB (Gama-Castro et al., 2016) and EcoCyc (Keseler et al., 2017) databases (Figure 4B), suggesting a certain degree of degeneracy for the recognition of constitutive promoters in *E. coli*. Thus, it has allowed us to identify a consensus for exogenous promoter recognition in *E. coli*, which can be an important resource for defining host-dependent constraints in functional metagenomics. Yet, it is possible that promoters that do not match the known consensus for RpoD could be reconginzed by alternative sigma factors, but this need to be further exploited in the future.

A seminal study in functional metagenomics provided by Gabor et al. (2004), estimated on a theoretical basis that 40% of the enzymatic activities present in a soil metagenomic library could be readily accessed using *E. coli* as a host in an independent gene expression mode. This prediction implies that at least 40% of the metagenomic promoters would also be recognized by *E. coli*. Contrastingly, recent empirical studies on *E. coli* and other hosts have shown that functional expression faces a myriad of challenges (Bernstein et al., 2007; Ekkers et al., 2012; Vester et al., 2015), reflecting significantly lower rates than the proposed by Gabor and collaborators (Gabor et al., 2004). In agreement with those studies, our work stresses the gap between theoretical estimations and experimental results, as we have observed only a small portion of the whole set of promoters is accessible for *E. coli* in metagenomics libraries (~1% of the clones assayed displayed detectable fluorescence in the plates)–in contrast to the previously predicted enzymatic activities recovery rate (~40%) (Gabor et al., 2004). Thus, we remark the importance of generation predictions on a combination of both experimental and computational data.

### Intrinsic challenges in functional metagenomic studies for promoter exploration

In order to address the constraints underlying our observations and predictions, we have selected some caveats raised during this study, which are intrinsic to functional metagenomics and regulatory studies. Firstly, functional metagenomics investigates a system—bacterial community—based on its genetic parts—metagenomic fragments—, thus it is limited to provide blurred (and somewhat biased) depiction of the whole—e.g., some promoters observed as constitutive might be repressed by the structural conformation of bacterial chromatin in the original organism (Dillon and Dorman, 2010), but not in the plasmidial context in the host. Secondly, the metagenomic host will always bias the results as it filters biological information according to its own molecular machinery (Guazzaroni et al., 2015; Lam et al., 2015; Alves Ld et al., 2017)—e.g., a promoter might be considered constitutive when its exogenous repressor is not expressed in the host. Another potential limitation of the strategy used here, is that the direct cloning of DNA fragments and screening for fluorescent clones would be biased toward the identification of promoters located near the fluorescent reporter. Yet, since we were able to identify promoters located more than 1 kb away from the reporter gene, this potential limitation would not be a concerning issue here. Lastly, the line between constitutive and regulated promoters has become rather arbitrary among studies as it usually relies on the experimental design and concepts adopted by each research group—e.g., some authors consider constitutive bacterial promoters as those that are active *in vivo* in all circumstances, while others define them as the promoters recognized *in vitro* by RNA polymerase RpoD holoenzyme alone in the absence of additional regulatory proteins (Shimada et al., 2014).

## Conclusions

In summary, we have focused in integrating experimental and *in silico* approaches to exploit the regulatory diversity from metagenomics DNA fragments by prospecting and characterizing novel promoter sequences in *E. coli*. From this, we were able to identify novel constitutive promoters using real-sized metagenomic DNA fragments, and a further dissection of individual clones allowed us to demonstrate that a number of internal promoters can be recognized by the host to drive gene expression *in vivo*. Further studies could be applied to exploit which type of sigma factors are contributing for the expression of the identifiable active promoter fragments. Despite the intrinsic limitations previously described, our strategy can be further optimized by high-throughput studies, which will be essential for expanding our current estimations into a more holistic landscape. Finally, we highlight that this work should be also useful for the applied sciences, expanding the current biotechnological toolbox through the discovery and characterisation of novel regulatory features.

## Data availability

The nucleotide sequences obtained for the plasmid inserts have been deposited in the GenBank database under the Accession numbers (KY939589 to KY939598), which are also shown in Table 2.

## Author contributions

CW, LA, M-EG, and RS-R: designed the experiments; CW and LA: performed the experiments; CW: analyzed the data; CW and RS-R: prepared the figures. CW and M-EG wrote the manuscript. All authors reviewed the manuscript.

### Conflict of interest statement

The authors declare that the research was conducted in the absence of any commercial or financial relationships that could be construed as a potential conflict of interest.
