# Supplementary material for: Mining Novel Constitutive Promoter Elements in Soil Metagenomic Libraries in Escherichia coli
Source: Front Microbiol. 2018 Jun 20;9:1344. doi: 10.3389/fmicb.2018.01344 (PMC6019500; doi:10.3389/fmicb.2018.01344)
Supplement: Supplementary file 1 [file Data_Sheet_1.pdf]

Supplementary Material

Mining novel constitutive promoter elements in soil metagenomic libraries in *Escherichia coli*

Cauã Antunes Westmann[1], Luana de Fátima Alves[2,3], Rafael Silva-Rocha[1], María-Eugenia Guazzaroni[2*]

*** Correspondence:**
María-Eugenia Guazzaroni
meguazzaroni@ffclrp.usp.br

Supplementary Figures

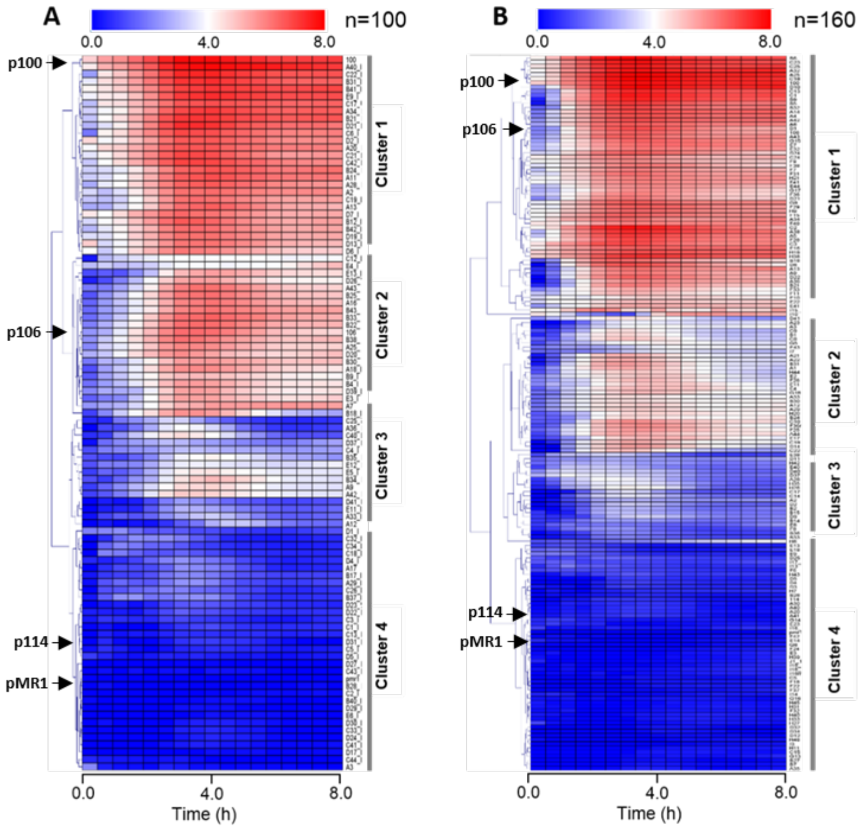

Figure S1. Hierarchical representation of metaconstitutomes for both metagenomic libraries highlighting expression trends as clusters. Fluorescence time-lapse dynamics were measured during 8 hours for each clone and represented as heat maps. Promoter activities (calculated as GFP/OD_{600}) were normalised by the negative control (*E. coli* DH10B harbouring empty pMR1) and transformed to log2 scale in order to facilitate the visualisation of subtle activities. Positive controls (p100, p106 and p114 - strong, medium and low expression, respectively) and negative control (pMR1) expression profiles are indicated by black arrows at the left side of the heatmap. Data are representative of three independent experiments. A) Dendrogram for USP3 metagenomic library composed by 100 fluorescent clones. B) Dendrogram for USP1 metagenomic library composed by 160 fluorescent clones.

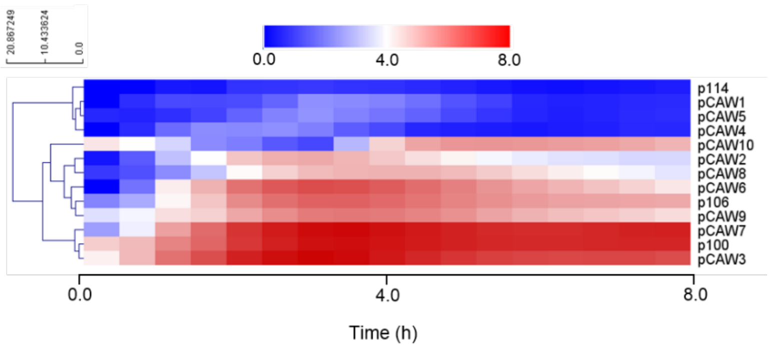

Figure S2. Expression profiles for the ten selected clones (pCAW1-pCAW10). Fluorescence time-lapse dynamics were measured during 8 hours for each clone and represented as heat maps. Promoter activities (calculated as GFP/OD600) were normalised by negative control (E. coli DH10B harbouring empty pMR1) and transformed to log2 scale in order to facilitate the visualisation of subtle activities. Data are representative of three independent experiments. Clones p100, p106 and p114 are positive controls for different promoter strengths, representing strong, medium and low expression, respectively. Hierarchical clustering of the selected clones according to their expression profiles.

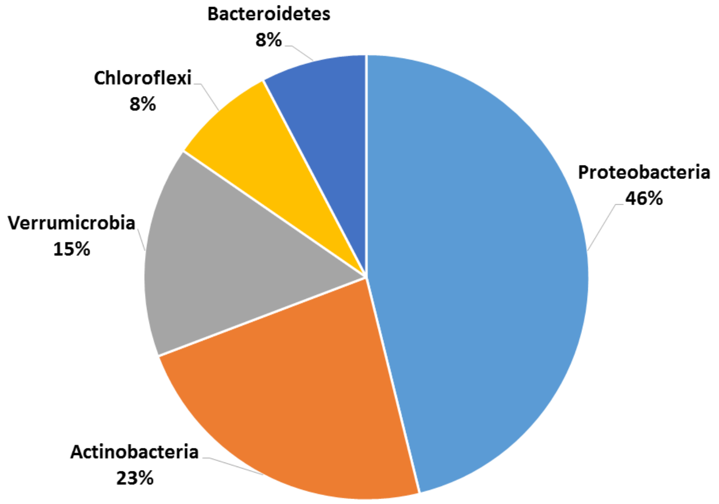

Figure S3. Abundance of microbial phyla with recognizable regulatory sequences in *E. coli* DH10B. The ten sequenced metagenomic fragments (pCAW1-pCAW10) were submitted to the *PhylopythiaS Web Server* and the assigned taxonomic origins were used for identifying the potential set of phyla recognizable by *E. coli* regarding exogenous promoter sequences.

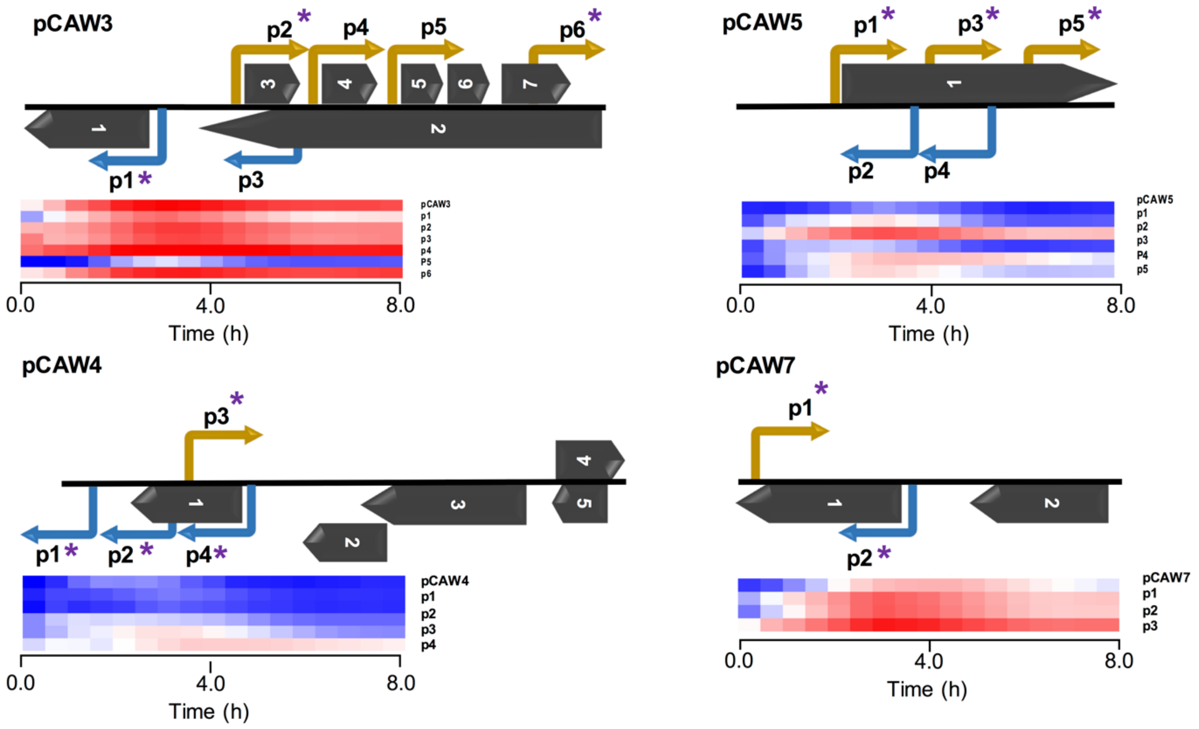

Figure S4. Schematic representation of the supplementary set of sequenced contigs showing predicted ORFs and validated/characterised promoters used in this work. Each contig is identified on the far left of each subfigure. Promoters are indicated by elbow-shaped arrows and name according to their relative position in the contig. Promoter directionality, regarding the leading and lagging strands, is represented by green and red colours, respectively. Asterisks over specific promoters indicate regulatory regions which were cross-validated by matching *in silico* predictions. Dark arrows represent predicted ORFs, according to their relative positions in each contig (see Table 2 for more information). Beneath each metagenomic insert, there is a heat map cluster representing the whole set of promoter activities measured during 8-hours fluorescence assays. A colour scale for all heat maps is provided in the first figure. The first line of each cluster shows the original expression profile initially measured for each metagenomic insert. All other lines represent expression activities from de novo experimentally validated promoters within each contig. The second line of each cluster represents the endogenous promoter showing the most similar activity with respect to the original expression profile for each contig. All expression profiles are properly identified at the most rightmost side of each line, following their respective contig/promoter name.

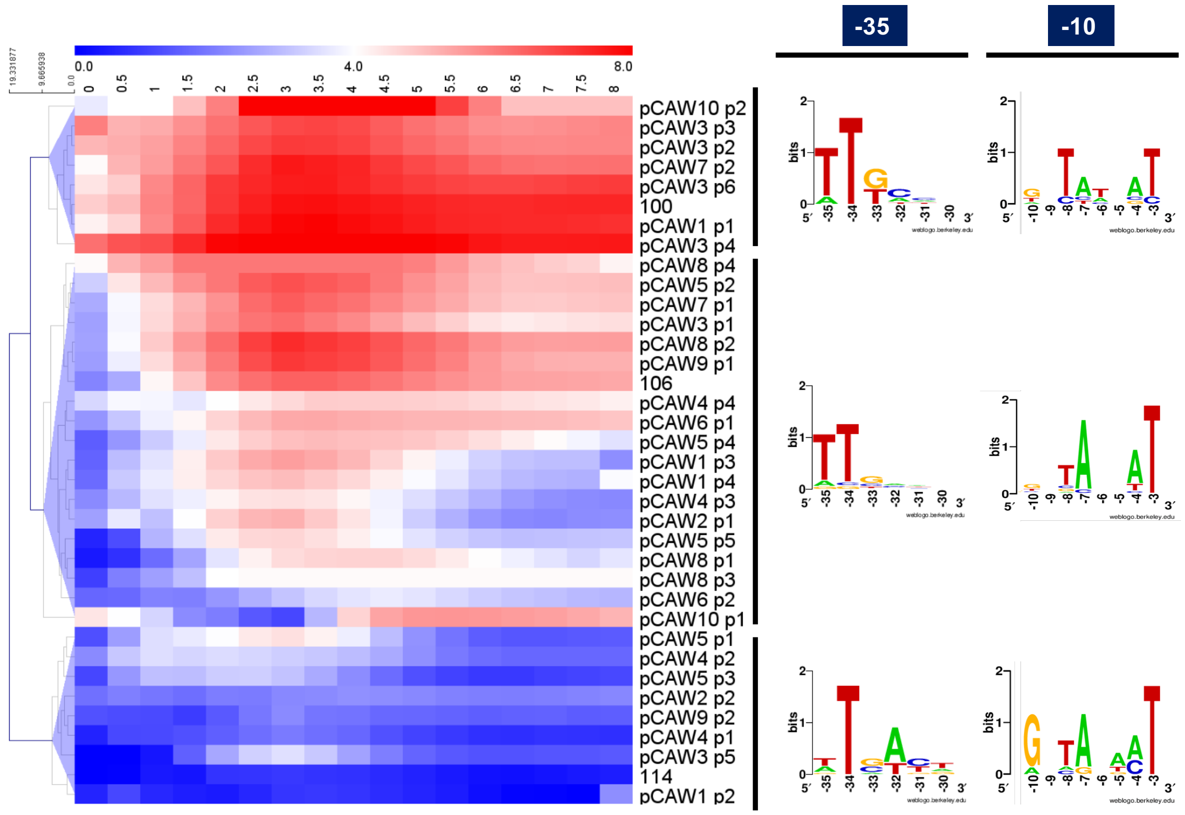

Figure S5. Consensus sequences for hierarchically clustered sets of experimentally validated promoters. Fluorescence time-lapse dynamics were measured during 8 hours for each clone and represented as heat maps. Promoter activities (calculated as GFP/OD600) were normalized by negative control (*E. coli* DH10B harboring empty pMR1) and transformed to log2 scale in order to facilitate the visualization of subtle activities. Data are representative of three independent experiments. Clones p100, p106 and p114 are positive controls for different promoter strengths, representing strong, medium and low expression strengths, respectively. All thirty-three experimentally validated promoters were organized by a hierarchical clustering method, revealing three general categories: strong (top), medium (middle) and weak (bottom) promoters. For each category, its respective set of promoter sequences was aligned using ClustalW (http://www.genome.jp/tools-bin/clustalw) and subjected to the *WebLogo* platform (https://weblogo.berkeley.edu/logo.cgi) for the generation of consensus sequences (right side).

able S1: Experimentally validated metagenomic promoters found in this study

Clone_Sample	Promoter ID	Sequence	Orientation	*In silico* validation
	p1	TACCGGTAACGACTTAGATGGGAGGCCGACACTGTACAACGTCGGTGTGTAGTTGG	Reverse	Yes
	p2	ACGGGATTCTATTGACTGCGGCTGCGGCTGTCAACAGTCAAAATTCGGTAATCGGCGCCGTGA	Forward	Yes
	p3	TTGCACTCGTCCGACAAAACTGCACCAACTACCGGCATTGATTAGAGTTTTGAAAATAGAGTTTAACCACGAT	Forward	No
**pCAW1**	p4	GTTGATCGGTGAGATTGGCCGCATCACCGCGGCTGAGGCGCGCGCGCTTGGCTACACGAACGTCTA	Forward	Yes
	p1	TTGCTCACCATACCAACCTCCCTTGCGAATTTTAATTAAGGCTGAATTCAAGTGGAT	Forward	Yes
**pCAW2**	p2	AGCGCATTCAATGACCTGTTCAACGATGTCCCGTCCTCTCGAAAAACTTTCGCCGGTCGGTTCGCGACAATTTCGAAGGCCAGAATGGATATCTAATGACCGT	Forward	Yes
	p1	ATCGTCACCTCCACAAAGAGCGACTCGCTGTATACCGTTGGCATGCTAGCTTTATCTGT	Reverse	Yes
	p2	CTAAGCACCTTCGGTAGTTTCCTGGAACGAAGCCGCTGAAATCCAGCTCTGCGTACCCAGTGAAGCC	Forward	Yes
	p3	GCGCGCAGGCCAGATCGTTAGCCTGAGGGAAGTGAGAGAAGAAAACTCGATCCTCCGCAGGAACGATAAGAAAC	Reverse	No
	p4	GGTAAACTTCCTGTATCTCGTTCACAACATATTCCTGAACCGCGAATGGTCCTTTGA	Forward	No
	p5	CAGATCGGTTGTCCTTTGTCTACCTTCTGACCATCTTTCACCAGCAGTGTAGCTCCGTAAGGAACGTTGTTGGTGA	Forward	No
**pCAW3**	p6	CGCGTCCAACAGTTGTTTCAATCAGTTTTCTTTTCAACTCACCACCTTCACGCACTATCGTTTTTACTTTGATGTGTGCGTGCAGGTCAACCTGTG	Forward	Yes
	p1	TTCTGCATTGGCTACAGCAGGAACTGCGCCGCCGCCAGCGCGCTGGCGACGCTGGGATGGTACCCTACCCT	Reverse	Yes
	p2	ACCCGGTCGATCCGGTCTGCACAGACCGCTACACCTATCAGCCAGCCATACCAGCCGAGGGGTTTGGAGTCGGCCTGACCACGT	Reverse	Yes
	p3	ATACGTGGTCAGGCCGACTCCAAACCCCTCGGCTGGTATGGCTGGCTGATAGGTGTAGCGGTCTGTGCAGACCGGATCGACCGGGTTC	Forward	Yes
**pCAW4**	p4	GGCAGCATCCATGCATCATTCCTCGGTAAAAGCCAGCCAGTAGGGGGTATGCCGTCTTGGAGTTTCACGACGT	Reverse	Yes
	p1	ACCGGGCAGGACCGTCCCAAGCCAAAATATCCCGGCATCCCGGTGACCTGTAACGGCAATCAACTCGTCGCCCAATACGTTGA	Forward	Yes
	p2	GAGCATCCCGTCCTGGATATTCATCCCGGGATTGAGGGTGAGCTCGTTGACCTTGCGTAAAATAATCCCTTGAT	Reverse	No
	p3	TCCCGGGATGAATATCCAGGACGGGATGCTCACCACCCATTCGGAGCGAACCTACCTGGCGCCG	Forward	Yes
	p4	AAGTCAGGATTGGTTCATTGAAATTGTTGCGGCGGGCCACGACGCCGTTCATGTGATGTTCCTGGTTTTGCACTGGGCCGAGCA	Reverse	No
**pCAW5**	p5	CGCCGCAACAATTTCAATGAACCAATCCTGACTTTCCTAAGCCGCGCCTACGAAGAATTCGGAAATCTCACCGGTCGCTATTACGG	Forward	Yes
	p1	CCCGACTCCTTTGAAAGTATTCTTTTCCTGGTTTAGCATTGGCGCTCAATCATTTGGCGGCGGACCGTCCACCCTGCAACTAATTCAGAAC	Forward	Yes
**pCAW6**	p2	ATCGGCCGGAGCAGAAGAATCGAGCAGGTCAGGGGTTTCCGAGGTCAGTTTAGGTCACTCCTTTCGGTG	Reverse	No
	p1	GTGGATCGTTTGGGTTATATTACCCTCAAAAAGGTTCGCAAACGCCCAATTGCCGTGTAACACGATATCAGGAGTATT	Forward	Yes
**pCAW7**	p2	ACGATCTACTTCTCGTTTTGCCTTCTTTTGTCGCTACACTCCACAATCGGCTTAAGCCAGAGCATACCAACAGACCGGGTAGTTAACTGGAAACTA	Reverse	Yes

pCAW8	p1	CTCTCGTCTTGCTCACCATACCAACCTCCCTTGCGAATTTTAATTAAGGCTGAATTCAAGTGGATCCCTATGATGGGGGTACAC	Forward	No
	p2	CCCTCGCACTTGTGCACTGGTGCCGGTGAAATCGAAGATGAGCGACGAGCCCCGGACAGTCACGGCGACGGCTAGCTTCACCGGCTGGTCGA	Reverse	Yes
	p3	ATCTTGTTCGCGGGCTTTTCCATTCGGTGGCTAAGCGCTATAGTTCGGCCCTATAGGAGAACATGCCA	Reverse	No
	p4	GCAAATGCTCCTTCTTCAATTCTATCCGCGCCTATGATCCCACCCGACAAATAGAACATAGACAAATTCGCGC	Forward	Yes
pCAW9	p1	CTTAATTTCTCCTCTTTAATTCTAGGTACCCGGGGATCGCGATCGCAAGGATCATCGCTATGATGCCATGGGCTTCATGAA	Forward	Yes
	p2	TGGCATCATAGCGATGATCCTTGCGATCGCGATCCCCGGGTACCTAGAATTAAAGAGGAGA	Reverse	Yes
pCAW10	p1	CATTTTTCGTTCAGGTTGCGTGCGCTTCGGCAGGCTCAGTGAGAACGAAGGCACCGATTGGTATGGA	Forward	Yes
	p2	GCACGCAAGGTTTCGCTATTGTGTGATTGACGCGGGTTCCGGCTTCGCCGGCCTGTCGCCTCAACTCAGTCCGACCAGCGACAATGCG	Reverse	No
